# Uneasy lies the head that wears a crown: How celebrity CEOs affect corporate social responsibility—An empirical study of Chinese listed firms

**DOI:** 10.1016/j.heliyon.2024.e41489

**Published:** 2024-12-24

**Authors:** Mengxi Niu

**Affiliations:** a School of International Culture and Communication, Communication University of Zhejiang, China; b Center for UK Studies, Communication University of Zhejiang, China

**Keywords:** Celebrity CEOs, Identity theory, Impression management, Corporate social responsibility (CSR)

## Abstract

This study examines the influence of celebrity Chief Executive Officers (CEOs) on corporate social responsibility (CSR) in Chinese listed firms in the A-share market from 2009 to 2020. We employ a multi-theoretical approach, drawing on upper echelons, impression management, and identity theories to explore several aspects of this topic. We find that celebrity CEOs are associated with better CSR engagement, suggesting that celebrity CEOs are likely to utilize CSR to strategically manage their impressions. We then explore four boundary conditions in the relationship between celebrity CEOs and CSR, and how celebrity identity mechanisms moderate this relationship. We find that CEOs' age, gender, and degree of media attention can strengthen the celebrity CEOs–CSR relationship, explaining the boundary conditions influencing the level of celebrity CEOs' impression management motivation. Additionally, environmental dynamism does not influence the association between celebrity CEOs and CSR performance. Our study provides new insights into the understanding of CSR antecedents, particularly from the perspective of CEOs’ psychological motivation, and contributes to formulating future CEO and CSR strategies.

## 1 Introduction

Chief Executive Officers (CEOs), regarded as beacons and pilots for corporate development, are essential to organisations. In recent years, outstanding business achievements and wide media attention have resulted in an increasing number of CEOs taking centre stage and being crowned celebrities. Simultaneously, many celebrity CEOs worldwide, such as the founder and chairman of the Chinese technology company Xiaomi Corp Lei Jun, Meta CEO Mark Zuckerberg, Amazon founder Jeff Bezos, and Tesla CEO Elon Musk, are focusing on corporate social responsibility (CSR), actively taking responsibility in education, medicine, technology, innovation, and so on.

Celebrity CEOs are a product of the media [1], as the media overly attributes past positive firm performance to individual CEOs rather than situational factors [2]. This ‘attribution bias’ not only triggers attention to celebrity CEOs but also leads to higher expectations of them achieving equivalent or even better corporate performance in the future. Additionally, over time, society has created new demands and aspirations for celebrity CEOs. For instance, they are expected to shoulder extensive social responsibilities and ensure the well-being of wider stakeholders under circumstances such as pandemics, unstable geopolitical situations, and climate change. For celebrity CEOs, these social expectations constantly merge with their understanding of themselves, hoping that their role presentation will conform to the public's expectations and lead to positive feedback. The identity pressure of ‘who am I’ and ‘what do I do’ causes celebrity CEOs to have strong impression management motives to protect and maintain their celebrity identity.

CEOs' participation in CSR activities is common and popular nowadays. Compared to non-celebrity CEOs, why are celebrity CEOs, with honour and halo, actively engaging in CSR activities? Are their CSR initiatives motivated by their celebrity identities? Although the CSR antecedents of corporate executives have attracted the attention of many scholars, few studies have investigated how CEOs' intrinsic need motivation might affect CSR participation. This omission is problematic, because motivations are generated by needs, and individuals with different identities may have different needs. Celebrity CEOs, as new and popular identity, are indulged with more self-esteem and self-efficacy (Burke & Stetes, 1999), and are likely to internalise the public's expectations of them[3]. Meanwhile, given the importance of CEOs in CSR decision-making in firm strategies [4], investigating CEOs' motivations for participating in CSR activities could provide useful insights and enrich the understanding of the antecedents of CSR engagement. Given the importance of CEOs in CSR decision-making in firm strategies [4], investigating CEOs' motivations for participating in CSR activities is imperative.

Upper echelons theory posits that various firm strategies and outcomes, including social performance policies [5], are significantly influenced by firm executives, particularly CEOs [6]. Following this line of research, some scholars have affirmed that corporate executives' attributes and values can force them to engage in CSR activities (e.g. Ref. [5,7]). Others have suggested that executives' self-serving motivation may also affect their CSR participation (e.g. Ref. [8,9]). Examining CSR antecedents from the perspective of corporate executives' self-serving motivation is practical and interesting, because motivation is generated by needs which vary among individuals under different situations and identities. Moreover, it enriches the understanding of CSR antecedents. This emerging literature stream has shown that engaging in CSR activities can satisfy CEOs' self-serving motives under different identities. These motivations include emotional wealth for CEOs in family firms [10] and hometown affection for CEOs in startups [11]. However, to the best of our knowledge, the implications of CEOs' self-serving motivations for CSR decisions have not been extended to the identity context in which celebrity CEOs, as a new identity category, may influence firms’ CSR participation.

We draw on prior research on celebrity CEOs to the study of CSR by investigating two interrelated research questions. First, we explore the mechanisms by which celebrity CEOs affect a firm's CSR participation, trying to answer why celebrity CEOs actively engage in CSR activities. Some scholars have recently examined the association between celebrity CEOs and CSR activities (e.g. Ref. [3]; Solomon & Bendickson, 2022), affirming that celebrity CEOs may engage more in CSR activities than non-celebrity CEOs owing to the unique social pressures generated by high visibility and status protection. Although these results provide some theoretical support to our investigations, we believe further research is required to examine the mechanisms between celebrity CEOs and CSR activities based on identity theory, particularly within the Chinese context. Theoretically, CSR engagement is regarded as an effective tool in impression management [9]. Most previous studies have focused on the use of CSR reports to improve corporate image (e.g. Ref. [12,13]). However, the demand for impression management can also be seen at the micro level, extending to corporate managers to satisfy the self-interested motives of management (Zhang et al., 2010). CSR participation may relate to managers' self-serving motivations [14].

Second, we examine how the contingent factors underpinning the celebrity CEO–CSR relationship could be weakened or strengthened to clarify circumstances that could affect celebrity CEOs' motivation. Prior research has discussed the external environmental factors that may enhance celebrity CEOs' engagement in CSR activities [3]. However, the extrinsic environment is not limited to competition and firm performance; media should also be a vital factor because it plays a central role in the process by broadcasting carefully controlled information about celebrities that embellishes both the extent of their achievement and the attractiveness of their identities [1,15]. Additionally, individual characteristics may affect one's perceptions of a threat to reputation. Research has affirmed that celebrities may form a self-concept consistent with the meaning assigned by society to them during their identification process [16] and internalise the public's expectations [3]. Thus, examining how celebrities' characteristics influence their CSR practices is interesting.

We test our hypotheses using a longitudinal dataset of Chinese listed firms in the A-share market from 2009 to 2020, revealing substantial support. This study makes several contributions. First, by demonstrating how celebrity CEOs affect CSR activities, we advance research on CSR antecedents, particularly CEOs' psychological motivation and understanding of why firms' CSR engagement varies. Second, it reveals some boundary conditions between the celebrity CEOs–CSR associations, providing an in-depth understanding of the circumstances reinforcing celebrity CEOs' impression management motivation. Notably, it enriches the CSR literature by linking upper echelons theory, identity theory, and impression management research to CSR studies, contributing to the scholarly understanding of CEOs’ influence on corporate social strategic decisions.

The remainder of this article is organised as follows. The theoretical orientations and hypotheses section provides an overview of key theoretical perspectives, discusses the existing literature, and develops the hypotheses. The research design section describes the data, measurements, and research model. The results section reports the main findings and the study's robustness tests. Finally, the discussion section discusses the findings. The final section concludes the study, highlights the implications, and presents the study's limitations.

## 2 Theoretical orientations and hypotheses

### 2.1 Impression management of celebrity CEOs

In his book *The Presentation of Self in Everyday life,* Erving Goffman [17] likened the world to a big stage and every individual to an actor on the stage of life. Goffman suggested that every role carries expectations. People hope that the audience will take their performance seriously and that their performance will meet the audience's expectations. Therefore, to align their impressions with others' expectations, individuals tend to manipulate and influence impressions using impression management via social interactions.

Impression management is a goal-oriented process intended to create or protect a desired perceived image [18]. Individuals strive to influence perceptions by creating desirable images and avoiding undesirable ones (Staw, 1991). Impression management involves two subprocesses: impression motivation and construction. Impression motivation involves the desire to create impressions in the minds of others. This is because creating good impressions can maximise the likelihood that one will obtain desired social outcomes (e.g. power and salary increments), maintain and facilitate self-esteem, and develop desired identities [19]. Once such motivations are developed, individuals may determine the suitable impression management tactics that should be adopted in their particular situations. This is known as the impression construction process.

We argue that their celebrity identity may result in celebrity CEOs having stronger impression management motivations than non-celebrity CEOs. Generally, celebrity CEOs face two types of pressures: identity maintenance and identity recognition.

#### 2.1.1 Identity maintenance pressure of celebrity CEOs

Celebrity identity not only provides advantages for celebrity CEOs but is also considered a highly beneficial strategy for companies [20]. For example, in addition to increasing CEOs' incomes and providing increased legitimacy and greater leadership power to CEOs, celebrity identity can also transform corporate reputation into a valuable strategic resource in enterprise competition. From the perspective of Maslow's hierarchy of needs theory, once individuals' basic needs are met, they pursue higher levels of needs such as self-esteem and self-fulfilment. Celebrity CEOs tend to gain praise, respect, and obedience when they receive active public attention and positive emotional feedback, thereby greatly satisfying their self-esteem and self-realisation needs. Thus, celebrity status is significantly useful and valuable for celebrity CEOs who are aware of their celebrity identity.

Despite its advantages, celebrity status is temporary [21]. Celebrity CEOs, who are often in the spotlight, are scrutinised by the media and public, magnifying firms' behaviours and judgement by media watchdogs. Therefore, celebrity CEOs must be mindful of their behaviour and firms' behaviours. When celebrity CEOs face difficult situations, their problematic behaviours can trigger negative perceptions and impressions of their identity among the public. Eliminating negative impressions is significantly difficult than establishing positive ones; the public's ‘knee-jerk reaction’ may link one negative impression with other negative associations of firms ([22]:250). Additionally, there exists a marginally increasing effect of negative information [23], indicating that negative information may further trigger a larger negative impact. Therefore, to some extent, every minor improper action can suddenly take away CEOs' previously glorified identity and popularity.

#### 2.1.2 Identity recognition pressure of celebrity CEOs

Given that roles are a prerequisite for identity and identity equals role identity [24,25], the answer to ‘who am I’ and ‘what do I do’—basic answers to identity—cannot be separated from the role played by individuals. Role identity is the cognition and definition of oneself and refers to individuals' representative behaviour in a specific context derived from others' needs and expectations [26].

Although management theorists have not explicitly proposed the specific qualities, behaviours, and goals celebrity CEOs should possess, Mintzberg [27] suggested 10 key roles that managers should play in firms—leaders, disseminators, spokespersons, and so on. As CEOs are the pilot and decision-maker in firms, their role expectations should be diverse, dimensional, and far-reaching than other managers. They should not only be responsible for corporate strategic development and financial performance but also balance the relationship between business and social responsibility. For instance, *Morning Consult* conducted a survey of 11,000 respondents (including consumers, financial decision-makers, and political activists), releasing a report in 2020 entitled ‘*Bigger Than the Boardroom—The Role CEOs are Expected to Play Today*’.^1^ The survey revealed that the public believes that CEOs should shoulder more social responsibility and ensure the well-being of employees and customers. In reality, an increasing number of CEOs are demonstrating their CSR determinations and achievements via media platforms; many win top media prizes for their outstanding achievements and contributions to CSR. This suggests that CSR is becoming a vital role expectation for CEOs.

The aforementioned role expectations of celebrity CEOs are reinforced by the media in two ways. First, celebrity CEOs are expected to take responsibility for corporate financial performance, stemming from the media's ‘attribution bias’ towards celebrity CEOs. The media overly attributes previous positive corporate outcomes to individual CEOs and spreads this logic to the public through media reports [2]. When providing information to the public, the media also manipulates their ideas and even influences or shapes their views on corporate attributes and behaviours [2]. Consequently, the public believes in and agrees with the media's attribution logic towards celebrity CEOs, expecting celebrity CEOs to take responsibility for firms' financial performance. Second, celebrity CEOs are expected to ensure the well-being of various stakeholders. In China, society is the stage for entrepreneurs to showcase their talents; only entrepreneurs who sincerely repay society and fulfil their social responsibilities can be truly recognised and meet the requirements of the times. Thus, Chinese celebrity CEOs, using their powerful influence, should be role models for CSR development.

Identity theory indicates that the core of identity involves categorising oneself into a certain role and integrating role-related meanings and expectations into oneself [28]. These meanings and expectations form a set of standards to guide individual behaviours [29], which should align with the expectations assigned to their roles [30]. During socialisation and individuals' assumption of new roles, such as CEOs, individuals tend to negotiate ‘who I am’ in the face of ‘who I am expected to be’ (Felix et al., 2023). However, identification is a relatively stable understanding of a specific role by oneself [31]; individuals may receive evaluations of their role-playing during social interactions. Identification has an automatic control system that reduces identification inconsistency, aiding individuals in modifying their behaviours and achieving consistency both internally and externally (Burke & Reitzes, 1991). Celebrity CEOs constantly internalise expectations in their interactions with the public, hoping that their role performance will meet expectations and result in positive evaluations. Individuals are motivated to maintain and increase their sense of self-worth and maximise social acceptance. They prefer to receive feedback about their positive attributes and tend to improve their self-esteem whenever possible [32]. Thus, celebrity CEOs face heavy identification pressure.

In summary, when faced with both identity maintenance and recognition pressures, celebrity CEOs are more likely to have strong impression management motivations to protect and maintain their celebrity identity.

### 2.2 Celebrity CEOs’ CSR participation

Following the discussion on celebrity CEOs' impression management motivations, this section delves into impression construction, which explains how individuals manipulate others’ impressions. We argue that celebrity CEOs tend to construct impressions through CSR activities for several reasons.

First, impression management involves ensuring external recognition. One way to ensure external recognition is to showcase correct and acceptable behaviours in specific contexts [33], which can be manifested through prosocial or helping behaviour [34]. CSR activities are a type of prosocial behaviour for corporations. Firms' managers are likely to expect that CSR would similarly help to bolster their status [35]. As economic activities are deeply embedded in social relations, firms can only survive in society by using its prosocial nature to respond to social concerns and fulfil corporate citizenship obligations. Therefore, as a key dimension of prosocial behaviour for firms, CSR can effectively ensure external recognition that reflects celebrity CEOs’ identity and roles.

Second, media attention to celebrity CEOs makes situations more common, further promoting helping behaviours. Research has confirmed that others' presence can be regarded as a social force that affects individuals' cognition, emotions, and behaviours. When individuals are conscious of being monitored by cameras, they are more likely to engage in helping behaviours [36]. However, being in others' presence does not need to be in the form of a ‘physical object’ (such as an actual camera); implying or imagining being in others' presence can have a similar impact on individuals' behaviour [37]. In the context of celebrity CEOs, the media is like a hidden monitor and a potential evaluation object in celebrity CEOs' minds. Therefore, the media can be considered an invisible presence of others for celebrity CEOs. Consequently, media presence may lead to increased helping behaviour in celebrity CEOs, which is reflected in CSR participation.

Third, CSR is an important strategy to meet stakeholders' expectations, aligning with the public's expectations of celebrity CEOs' role. Celebrity firms' social significance attracts high levels of stakeholder attention to the firms and their behaviors [38]. Stakeholders' perceptions of CSR derive from whether corporate behaviors ensure their well-being, which is vital for companies to build relationships with their stakeholders [39]. Additionally, CSR activities convey the message that stakeholders' needs and appeals are understood by firms. Owing to self-image building in a shared system of meaning and ethics [40], stakeholders' identification with firms can be extended to identification with celebrity CEOs' role. When engaging in CSR, CEOs feel satisfied with themselves and perceive themselves as socially important, consequently their self-perceived status increases [41]. More importantly, managers and leaders drive firms' strategies to send strong signals to stakeholders about their strategic importance in the market [42]. Moreover, CSR can be used as an alternative method to compensate for a decrease in firms' financial performance. During financial difficulties, firms can, to a certain extent, recover their image and reputation using the reputation capital accumulated through CSR [43]. Positive moral capital can build positive firm attributes that may compensate for performance decline. Hence, we posit that celebrity CEOs foster increased CSR engagement in firms.

Hypothesis 1 Celebrity CEOs and firms' engagement in CSR activities are positively related.

### 2.3 Moderating factors of the celebrity CEOs–CSR relationship

Based on our previous discussion, we explore whether certain contingency factors affect celebrity CEOs' motivation for impression management to protect their celebrity identity; this could influence celebrity CEO's participation in CSR activities. Leary and Kowalski [18] suggested that individuals' motivation for impression management depends on three conditions: 1) the stronger the relevance between the target audience's impression of individuals and individuals' impression management target, the stronger the motivation for impression management. The relevance of the impression management target is restricted by two factors: individual behaviour openness and individuals' dependence on the target audience; 2) the higher the value of individuals' impression management target, the stronger the motivation for impression management; and 3) the greater the gap between individuals' expected and perceived impression, the stronger the motivation for impression management [18,44]. Additionally, Wang and Li [45] highlighted that individual characteristics and external environmental factors influence impression management.

In line with previous arguments, we posit that celebrity CEOs' motivation for impression management can be affected by the aforementioned three conditions. When any of these factors changes, celebrity CEOs’ impression management desire is altered, ultimately affecting their CSR engagement.

Hence, according to prior scholars and insights from sample investigations, four contingency factors are discussed: CEOs' characteristics (including age and gender) and environmental conditions (including environmental dynamism and media attention). Identifying contingent factors allows us to further examine the core logic behind our main hypotheses, aiding the understanding of the factors that can alter celebrity CEOs’ motivation for impression management and how these factors strengthen the celebrity CEOs–CSR relationship.

### 2.4 Boundary effects of CEOs’ personal characteristics

We focused on CEOs’ age and gender as moderator variables in our research, based on the discoveries and insights from the sample data. We found that Chinese listed companies are male dominated; male CEOs account for approximately 90 % of the total number of CEOs. Moreover, the average age of CEOs in the sample is 49 years old; the oldest CEO is 78 years old.

The abovementioned age and gender distribution of Chinese CEOs is similar to that of CEOs worldwide. According to a 2019 report by the International Labour Organisation which surveyed approximately 13,000 companies across 70 countries, a significant ‘leaky pipeline’ phenomenon exists in corporate management, with the proportion of women decreasing as the management level increases. Over 78 % of the surveyed companies had male CEOs, with female CEOs predominantly found in small businesses. Meanwhile, according to the ‘Route to the Top 2021’ annual report on CEOs of listed companies in 24 countries and regions conducted by Heidrick and Struggles, the average age of newly appointed CEOs in 2021 was 49 years old.

Our observation of the data sample confirms that the characteristics of CEOs of Chinese listed companies in terms of age and gender are a microcosm of the international business environment. It also stimulates our interest in exploring the impact of age and gender factors on management decision-making issues and organisational performance.

Second, both age and gender are key variables worthy of attention and further exploration; their theoretical significance will be discussed in detail in the following sections.

#### 2.4.1 CEO age

Age is an important characteristic that can trigger individual differences in cognition, personality, and mental state. Age is not only an indispensable factor in psychological research but also a significant variable in exploring the relationship between corporate executives and corporate strategic decisions in management. Upper echelons theory suggests that corporate executives' behaviour differs at various ages. Furthermore, managers’ age can impact their decision-making ability and showcase their behavioural preferences.

We posit that age may increase the identity gap between celebrity CEOs' expected impression and the public's perceived impression during the impression management process. Age does not directly lead to this gap. However, decision-making behaviours and performance outcomes of celebrity CEOs caused by age can lead to an identity gap. Age can influence celebrity CEOs' cognitive abilities, resulting in rigid and conservative strategic decisions that may influence corporate performance. Particularly, the method of acquiring and explaining information varies with age group. Aging maybe accompanied by a decrease in strength and energy. Therefore, older managers may be less skilled in detailed information processing [46] and integration than younger managers [47]. Consequently, they are more likely to rely on rigid cognitive patterns already formed in firms.

Additionally, older corporate executives are less willing to invest in technological innovation. Given the uncertainty between innovation input and output in a time-consuming R&D process, older CEOs may believe that larger R&D investments may not necessarily yield increased benefits. Thus, older CEOs may prefer to reduce R&D investments during their upcoming departure to protect their position (Dechaw & Sloan, 1991), even though corporate innovation outcomes are highly related to positive financial performance.

On the other hand, the managerial signalling model (Prendergast & Stole, 1996) suggests that younger managers tend to showcase themselves, overestimate information, and exhibit overconfidence in decision-making, placing high value on personal beliefs and abilities. Therefore, younger CEOs engage in more aggressive risk-taking behaviours to demonstrate their management and operational capabilities, showcase their talent, and build external personal reputations.

In contrast to younger CEOs' boldness and courage, older CEOs tend to rely on past experience and industry standards to assess situations and make decisions while adhering to familiar business methods and paths. They opt for safer and more conservative investment strategies, reducing corporate investment activities and efficiency. Additionally, when executives encounter highly uncertain decision settings, they may draw from previous experiences and repeat the actions taken when faced with similar situations [48]. However, these overly conservative strategic choices could lead to less inefficient corporate investments, which would negatively impact corporate financial achievements. As investment analyst Don Bilson from Gordon Haskett noted in an interview with Barrons, ‘older CEOs are dangerous CEOs; they are the ones who cause stock price volatility’.

In summary, the aforementioned situations can cause fluctuations in corporate financial performance, which conflicts with the external expectations of celebrity CEOs responsible for producing positive corporate outcomes. Therefore, celebrity CEOs are more likely to manage their impressions through CSR to compensate for the impression gap caused by potentially negative corporate financial performance.

Hence, we posit that CEOs' age strengthens celebrity CEOs’ impact on CSR.

Hypothesis 2 The positive relationship between celebrity CEOs and firms' CSR engagement strengthens when CEOs are older.

#### 2.4.2 CEO gender

First, we suggest that perceptions of impression management targets vary with gender. Gender differences significantly impact self-presentation. Individuals’ value judgements for maintaining their identity and status differ with gender. Compared to females, males are more likely to have stronger desires and motivations for seeking status. This is because males are driven by reproductive interests for human evolution.

According to evolutionary psychology researchers, several adaptability problems and selective pressures have existed throughout human existence and reproduction. Humans have evolved into a special status-pursuing mode to address these issues and conform to evolutionary laws. Based on the theory of social status hierarchy [49], social animals have developed a strategy to compete for survival and reproduction resources under evolutionary selection pressure. Individuals' status hierarchy reflects their right to access resources, particularly scarce resources; the higher the individuals’ status, the more resources they can obtain. Meanwhile, in the reproduction process, women tend to invest more in parenting than men, allocating more time and energy to raise offspring; thus, women prefer men with a higher status who can afford sufficient resources to breed offspring. Hence, males have evolved into the psychological mechanism of pursuing status.

Notably, psychologist Pratto and colleagues [50] also affirmed that males' psychological mechanism of status pursuit applies to workplace situations, demonstrating that males treasure their status in the workplace more than females. In the context of celebrity CEOs, celebrity identity not only reflects higher social status but also manifests males’ status-pursuing mechanisms in the workplace. Therefore, male CEOs are more inclined than female CEOs to emphasise identity protection, which may further enhance their impression management motivation and CSR investment.

Second, we posit that gender may contribute to the differences between celebrity CEOs' self-perceptions and external impressions. Societal expectations of gender roles impact behaviour. Individuals internalise these societal projections of gender expectations, eventually transforming them into an inherent driving force that aligns their behaviour with societal gender cultural expectations [51]. Individuals should possess perceptions and beliefs about masculine and feminine roles and identify the ideal psychological structures associated with their respective gender groups. These beliefs can be manifested through behaviours, attitudes, and emotional expressions aligning with masculine or feminine traits. This gender-based role identification transcends mere behavioural states associated with biological sex, focusing more on the motivations, attitudes, values, and behaviours that individuals are expected to embody based on societal cultural expectations of masculinity and femininity. Society expects women to be more nurturing, friendly, sensitive, and adept at expressing emotions as they primarily assume the caregiver role. This gender cognition leads to the formation of gender stereotypes, in which women are perceived as communal and socially oriented (communal type), whereas men are viewed as competitive and achievement-oriented (agentic type) (Eagly & Mladinic, 1989). Role identities shape individuals’ expectations of normative behaviour, with gender differences being a crucial factor influencing behavioural normativity, thus affecting impression management strategies [52].

Therefore, according to role congruity [53], women are expected to conform to their gender role expectations. The gender of leaders may influence the consequent activities of the firms they lead [54]. Coupled with their inherent empathetic caregiving traits, women tend to focus on ‘soft’ issues such as CSR, thus contributing to more favourable decision-making regarding companies' CSR initiatives. Based on role congruity and gender stereotypes, male managers are less likely to focus on behaviours that contradict their role images. For instance, their contributions and attention to CSR-related affairs are generally lower than those of female managers.Coupled with their inherent empathetic caregiving traits, women tend to focus on ‘soft’ issues such as CSR, thus contributing to more favourable decision-making regarding companies' CSR initiatives. Based on role congruity and gender stereotypes, male managers are less likely to focus on behaviours that contradict their role images. For instance, their contributions and attention to CSR-related affairs are generally lower than those of female managers.

Hence, we posit that male CEOs strengthen celebrity CEOs’ impact on CSR.

Hypothesis 3 The positive relationship between celebrity CEOs and firms' CSR engagement strengthens when CEOs are males.

### 2.5 Boundary effects of the external environment

Business operations and development depend on the external operating environment. It not only provides the necessary conditions for companies' production and operational activities but also imposes constraints on them. Additionally, the external environment is a vital factor influencing impression management motivation [45]. Specifically, companies’ survival and development cannot be separated from two environments: operating and media.

Fluctuations in environmental factors can affect a company's strategy, operations, and performance. Misalignment with the environment can severely affect a company's resource acquisition, capability development, and performance improvement. Moreover, a high level of environmental uncertainty is not only an objective external existence but can also transform into uncertain individual perceptions which individuals cannot accurately predict [55]. Characteristics and changes in the environment inevitably affect the direction, content, and manner of organisational activities. As important participants and key coordinators in the interaction between companies and the environment, business managers are inevitably affected by changes in organisational outcomes. For example, a company's poor performance may lead to the CEO's replacement or management being burdened with a ‘bad’ reputation owing to corporate scandals and face the risk of dismissal, which can affect their future careers and personal wealth [56].

Additionally, the development of commerce has nurtured a close relationship between enterprises and the media. As New York Times journalist Barbosa stated after winning the Pulitzer Prize, ‘everything is closely related to business’. Business news has flourished with the increasing demand for business information. Enterprise news, as a focus of business news, was the subject of at least one-third of Pulitzer Prize-winning works in2016.^2^ Furthermore, the media not only serves as a vital information dissemination medium but is also vital to corporate governance through three mechanisms: market supervision, reputation, and market pressure [57]. Based on stakeholder theory, the reputation mechanism refers to the media's impact on a company's or management's reputation and image when collecting and reporting information, which affects corporate performance (Liu & McConnell, 2013).

#### 2.5.1 Environmental dynamism

We suggest that a dynamic environment increases the identity gap between celebrity CEOs' expected impressions and the public's perceived impression. Corporate development inherently depends on the external environment; the more changes and fluctuations in environmental elements, the higher the unpredictability and uncertainty in corporate strategies, operations, and performance. For a company, a high degree of environmental dynamism may lead to loss of key resources and information [58]. Moreover, environmental dynamism indicates a constant emergence of technological innovation, shortened product life cycle, unpredictable market demand, and competitive landscape fluctuations [59]. In such circumstances, corporate executives must prioritise market changes and promptly adjust products based on market monitoring. However, these behaviours may increase operating costs and affect financial performance.

Additionally, environmental uncertainty can be transferred into individuals' uncertain perceptions. Milliken [55] suggested that environmental uncertainty is a perception describing individuals who cannot accurately predict an organisation's environment. This individual perceived uncertainty includes individuals' inability to predict event occurrence, impact of the organisation's environment, and decision outcomes. For celebrity CEOs, a dynamic environment may reduce both their sense of control and confidence in business operations. This sense of insecurity and powerlessness may decrease their identification with their identity. Moreover, environmental fluctuations may reduce the evaluation of celebrity CEOs to some extent, which may also threaten their reputation. Therefore, celebrity CEOs are eager to participate in CSR activities to complement their positive reputations.

Moreover, environmental dynamism increases the dependence of corporate development on the resources and support provided by stakeholders; thus, obtaining external resources via social exchange is becoming particularly important [60]. Accordingly, CSR engagement is an efficient approach that can help strengthen communication and establish trusting relationships with stakeholders. It aims to seek potential market opportunities and fill the impression gap created by dynamic environments. Hence, we posit that environmental dynamism strengthens celebrity CEOs’ impact on CSR.

Hypothesis 4 The positive relationship between celebrity CEOs and firms' CSR engagement strengthens in dynamic environments.

#### 2.5.2 Media attention

First, we suggest that excessive media attention may enhance the transparency of celebrity CEOs' situations, amplifying the media's role in corporate governance and augmenting the pressure on celebrity CEOs to be supervised. As a sensegiver of moral legitimation, the media reflects commonly held assumptions and actively shapes public opinion [61]. Under media scrutiny, celebrity CEOs tend to enhance their self-awareness and conduct self-evaluations of their impressions on the public. This motivates celebrity CEOs to perform in accordance with socially acceptable norms to ensure identity recognition.

Second, media attention increases celebrity CEOs' perceptions of impression and identity gap. Social information processing theory suggests that the more frequently attribution logic is promoted, the more likely individuals are to accept it [62]. In the context of celebrity CEOs, excessive media attention may reinforce the understanding that celebrity CEOs are responsible for positive financial outcomes. Consequently, celebrity CEOs trust and internalise this attribution logic as their role identification. Additionally, while providing information to the audience, the media manipulate and shape the public's views on organisational attributes [2,20]. Thus, a high degree of media attention will also intensify the recognition of attribution logic by external audiences (including relatable corporate stakeholders). Therefore, public feedback may add to the burden of celebrity CEOs and deepen self-identification of their celebrity identity.

In summary, the more excessive the media attention, the greater the transparency of celebrity CEOs’ situations and the stronger the identity differences perceived by celebrity CEOs. Hence, we posit that celebrity CEOs tend to achieve better CSR performance to enhance their positive impressions and compensate for the impression gap.

Hypothesis 5 The positive relationship between celebrity CEOs and firms' CSR engagement strengthens under excessive media attention.

## 3 Research design

### 3.1 Data

We examined the sample observations from 2009 to 2020. Firstly, we considered that the global financial crisis in 2008 should have an impact on the strategic decisions and operations of certain companies worldwide. Thus, the data collected from 2009 onward is considered to be more robust. Secondly, CSR performance (*CSR*), as the dependent variable in this paper, we measured CSR performance (*CSR*) at year *t+1*; thus, the observation period for the entire sample is from 2009 to 2021. We employed CSR rating scores in *Hexun* CSR rating database to measure CSR performance, considering that the data from the *Hexun* CSR rating database suffered a significant missing data in 2022, thus, 2021 is the last year for us to obtain relatively complete CSR data during the writing.

The study's data were gathered from multiple sources. First, we identified six authoritative Chinese media that rewarded financial prizes for outstanding corporate leaders from 2009 to 2020, based on the information provided on their official websites. These awards include *China Economic Person of the Year* (awarded by China Central Television), *The Best CEO of Chinese Listed Companies* (awarded by Forbes China), *Top 10 Outstanding CEOs*, *Top 10 Growth CEOs* and *China CEO Person of the Year* (awarded by China Times), and *Top 10 Economic Person of the Year* (jointly awarded by Sina Finance, People's Daily, and Wu Xiaobo Channel). The selection process for these awards have been referred to in prior studies (i.e. [3,62,63]). Next, we obtained the name lists of all winners and verified the companies for which they work. To ensure the sample's reliability, we excluded firms with the following characteristics: 1) non-Chinese listed firms in the A-share market; 2) firms belonging to the financial industry (such as banks and insurance companies); 3) the prize winner is not a CEO (such as when the firm's chairman won the prize); 4) firms with abnormal operating and financial conditions at the end of the year (when the prize was awarded), marked with ST or ∗ST; 5) if the winning CEO experienced a change after winning the prize at the end of the observation year; and 6) missing vital CEOs' personal information.

Additionally, data on CSR performance were obtained from the *Hexun* database, a reputable CSR rating database in China and widely used in CSR research in the Chinese context. Information on CEOs' age and gender were mainly acquired from the ‘*Character Characteristics of Listed Companies*’ category in the China Stock Market Accounting Research database (*CSMAR*). Certain age and gender data were supplemented using firms' annual reports, firms' official websites, and *Baidu Search* (the world's leading Chinese search engine). The control variables were obtained from the *CSMAR*.

Our final sample included 251 firms and 2,848 firm-year observations. Owing to the time-lag effect on CSR performance, we measured CSR performance (*CSR*) at year *t+1*; thus, the observation period for the entire sample is from 2009 to 2021. Moreover, to avoid the impact of extremes, all continuous variables were winsorised at the 1 % and 99 % quantiles. Additionally, to eliminate multicollinearity between variables, all continuous variables were centralised.

### 3.2 Measures

#### 3.2.1 CSR performance

CSR performance (*CSR*) is the dependent variable. We employed a third-party rating method to measure CSR performance. This process involved evaluating sample companies’ CSR performance using the *Hexun* CSR rating database. Compared to the other two CSR measurement methods (i.e. content analysis and reputation index methods), the third-party rating method is more objective in assessing CSR performance and is applicable to research contexts involving Chinese listed firms. This method is also widely recognised as an authoritative measurement approach commonly used by mainstream Chinese scholars in related studies (e.g. Ref. [[64], [65], [66], [67], [68], [69]]). Third-party rating method was the usual way for foreign scholars in mainstream journals to measure CSR with third-party rating scores (e.g. MaWilliams & Siegel, 2000; [70]), namely the KLD social index score, which provides ratings of corporate social performance or CSP (a measure of corporate social responsibility). As originally suggested by Ullmann [71], Waddock and Graves [72] constructed KLD as an aggregate measure of corporate social performance.

Content analysis involves assessing the quality of social responsibility information disclosed in listed firms' annual and CSR reports. This method examines factors such as the number of words, lines, and comprehensiveness of the specific reports covered. However, this approach has limitations because it derives CSR commitments from corporate disclosures, leading to indirect content acquisition. Moreover, CSR disclosures in some companies' annual reports may not be standardised or may include missing information. Additionally, researchers’ subjective judgements may affect the objectivity and fairness of the content analysis process.

The reputation index method involves distributing surveys to respondents (such as company stakeholders) to gather evaluations of a company's CSR performance. However, this method cannot entirely eliminate subjective interference from respondents' attitudes towards the company, company size, industry competition, or other factors.

The evaluation system rates listed firms across five dimensions: ‘shareholder equity responsibility’, ‘employee responsibility’, ‘supplier, customer and consumer rights responsibility’, ‘environmental responsibility’, and ‘social responsibility contribution’. It assigns weights to these five dimensions based on the industry in which the company operates, establishing 13 secondary and 37 tertiary indicators. Finally, the company's CSR score for the year is calculated based on these scores and corresponding weights, with a maximum score of 100. A higher score indicates better CSR performance for the year.

Based on the above considerations and our research context, we chose the CSR rating data in *Hexun* database to measure CSR performance of the sample firms. Specifically, we used ‘*CSR Overall Rating Score’* to measure CSR performance (*CSR*) in our main research models. In the robustness checks discussed below, we replicated the main findings using ‘*CSR Overall Market Rating Score*’ to measure CSR performance (*CSR_MScore*).

#### 3.2.2 Celebrity CEOs

In this study, we define celebrity CEOs as those who are attributed by the media for their company's positive performance, particularly owing to their personal capabilities as CEOs, and are bestowed with celebrity status through awards and recognition. As the independent variable in our study, the celebrity CEOs (*Winner*) variable indicates whether the CEO of company *i* has won awards from the six authoritative Chinese media outlets in year *t*. Celebrity CEOs (*Winner*) was scaled using a binary dummy variable which takes the value of 1 if the CEOs won the prizes during 2019–2020; otherwise, it takes the value of 0.

#### 3.2.3 Moderating variables

The two moderators representing CEO characteristics are age and gender. CEO age (*CEO_Age*) was measured using celebrity CEOs’ physical age at year *t*. CEO gender was scaled using a binary dummy variable; *CEO_Male* takes the value of 1 if the CEO is male and is 0 otherwise.

The external environmental moderators measure both management and media environments. Environmental dynamism indicates the degree of instability in the external management environment of organisations [73]. Dynamic external environments inevitably cause fluctuations in firms' core business activities, which affects firms' sales profitability. To account for this, we adopted a practical approach employed by prior research using sales revenue to measure environmental dynamism (*ED*) [[74], [75], [76]]. Specifically, the sales revenue of the company in year *t*, *t-1*, *t-2*, *t-3,* and *t-4* is taken as the dependent variable, and *5*, *4*, *3*, *2,* and *1* are taken as the independent variable. Environmental dynamism (*ED*) was measured using the standard error of the regression coefficient of the obtained model divided by the mean value of the company's five-year sales.

For the media attention variable, given that our study is situated in the business field, business-related news is categorised as financial news, and reading news via the Internet is preferred [77], we focused on online financial news to acquire data relating to media news. Based on key measurement methods by Chinese scholars [78,79], we obtained online financial news data from the Chinese Research Data Services (*CNRDS*) database. The *CNRDS* database includes the top 20 mainstream online financial media outlets in China, such as *Hexun Website*, *Sina Finance*, *Tencent Finance*, *Phoenix Business*, *Financial Times Chinese.com*, and so on. Specifically, we scaled media attention (*MA*) by counting the total number of online financial news items with the name of company *i* appearing in news headlines in year *t.* We focused on news headlines because the layout of online news is different from other news forms. News titles include key information related to the news article and are first seen by audiences [80]. Therefore, displaying a firm's name in news headlines can better reflect the amount of media attention paid to the firm. In the robustness checks discussed below, we replicated the main findings using the total number of online financial news articles with the name of company *i* appearing in the news content in year *t* to measure media attention (*MA_Content*).

#### 3.2.4 Control variables

A firm's CSR engagement in the previous year was controlled for as it captures the firm's CSR history [81]. A firm's financial situation has been shown to affect its CSR engagement; the better a firm's financial status, the more finance it has to afford CSR expenditures [82]. Thus, firm financial performance, indicated by the leverage ratio (*Lev*) and cash flow ratio (*CF*), was controlled for. Firm size (*Size)* was measured using the natural logarithm of a firm's total assets. Additionally, we controlled for firm age (*F_Age*), state ownership (*State*), CEO tenure (*Tenure*), CEO duality (*Dual*), board size (*Boardsize*), and managerial shareholding proportion (*MShare*). Finally, we controlled for industry- and year-fixed effects.

### 3.3 Research model

We used a fixed-effects model to empirically test our first hypothesis on the association between celebrity CEOs (*Winner*) and CSR performance (*CSR*).

(1) $${\text{CSR}}_{i,t+1}=\beta_{0}+\beta_{1}{\text{Winner}}_{i,t}+\beta_{2}{\text{Controls}}_{i,t}+{\sum \text{Year}+\sum \text{Industry}+\varepsilon }_{i,t}$$

In Model (1), celebrity CEO (*Winner*), as the independent variable, is a dummy variable. The dependent variable, CSR performance (*CSR*), is a continuous variable.

Furthermore, to examine the moderating role of CEO characteristics based on the main effect model, we included the moderating variables CEO age (*CEO_Age*), CEO gender (*CEO_Male*), and their interactions with celebrity CEOs (*Winner*). This formed moderating effect Models (2) and (3) to empirically test hypotheses 2 and 3, respectively.

(2) $${\text{CSR}}_{i,t+1}=\beta_{0}+\beta_{1}{\text{Winner}}_{i,t}+\beta_{2\;}{\text{CEO}\_\text{Age}}_{i,t}+\beta_{3\;}{\text{Winner}}_{i,t}*{\text{CEO}\_\text{Male}}_{i,t}+\beta_{4\;}\text{Controls}+\sum \text{Year}+\sum \text{Industry}+\varepsilon_{i,t}$$

(3) $${\text{CSR}}_{i,t+1}=\beta_{0}+\beta_{1}{\text{Winner}}_{i,t}+\beta_{2\;}{\text{CEO}\_\text{Male}}_{i,t}+\beta_{3\;}{\text{Winner}}_{i,t}*{\text{CEO}\_\text{Age}}_{i,t}+\beta_{4\;}\text{Controls}+\sum \text{Year}+\sum \text{Industry}+\varepsilon_{i,t}$$

Additionally, to examine the moderating role of the two external environmental factors, based on the main effect model, we further included the moderating variables environmental dynamism (*ED*), media attention (*MA*), and their interactions with celebrity CEOs (*Winner*). This forms moderating effect Models (4) and (5) to empirically test hypotheses 4 and 5, respectively.

(4) $${\text{CSR}}_{i,t+1}=\beta_{0}+\beta_{1}{\text{Winner}}_{i,t}+\beta_{2\;}{\text{ED}}_{i,t}+\beta_{3\;}{\text{Winner}}_{i,t}*{\text{ED}}_{i,t}+\beta_{4\;}\text{Controls}+\sum \text{Year}+\sum \text{Industry}+\varepsilon_{i,t}$$

(5) $${\text{CSR}}_{i,t+1}=\beta_{0}+\beta_{1}{\text{Winner}}_{i,t}+\beta_{2\;}{\text{MA}}_{i,t}+\beta_{3\;}{\text{Winner}}_{i,t}*{\text{MA}}_{i,t}+\beta_{4\;}\text{Controls}+\sum \text{Year}+\sum \text{Industry}+\varepsilon_{i,t}$$

## 4 Results

### 4.1 Main regression results

Table 1 presents the descriptive statistics, and Table 2 shows the pairwise correlations among the variables in this study. Our main findings are presented in Table 3, which summarises the results of all hypotheses testing. Table 4 reports the robustness tests of celebrity CEOs and moderators on CSR performance. In Table 3, Model 1 includes all control variables and the main independent variable, celebrity CEOs (*Winner*). As shown in Model 1, the estimated coefficient on celebrity CEOs (*Winner*) is positive and significant (*β* = 3.827*, p* < 0.01), indicating that celebrity CEOs are positively related to CSR performance, thereby supporting Hypothesis 1.

**Table 1:** Descriptive statistics.

Variables	Obs	Mean	Median	Std.Dev.	Min	Max
**Dependent Variable**						
CSR	2542	30.406	25.750	18.639	−14.890	90.870
**Independent Variable**						
Winner	2721	0.141	0.000	0.348	0.000	1.000
**Moderating Variables**						
CEO_Age	2468	48.800	49.000	6.347	28.000	76.000
CEO_Male	2469	0.907	1.000	0.290	0.000	1.000
ED	2227	0.052	0.036	0.053	0.004	0.448
MA	2683	4.805	4.927	1.214	0.000	8.587
**Control Variables**						
Shareholder	2542	16.214	17.000	6.176	−10.720	28.190
Tenure	2467	5.675	5.000	3.688	1.000	18.000
Dual	2469	0.306	0.000	0.461	0.000	1.000
Size	2657	22.730	22.550	1.458	19.410	26.400
Lev	2657	0.425	0.428	0.208	0.027	0.906
CF	2657	0.062	0.055	0.076	−0.224	0.283
Boardsize	2657	2.139	2.197	0.195	1.609	2.708
State	2657	0.186	0.000	0.389	0.000	1.000
F_Age	2657	2.820	2.890	0.385	1.099	3.555
MShare	2599	0.136	0.0150	0.195	0.000	0.706
Year	2721	–	–	–	2009	2020
Industry	2721	–	–	–	A01	S90

**Table 2:** Correlation analysis.

Variable	1	2	3	4	5	6	7	8	9	10	11	12	13	14	15
1. Winner	1														
2. CSR	**0.135**	1													
3. CEO_Age	−0.004	0.012	1												
4. CEO_Male	0.003	−0.016	−0.033	1											
5. ED	−0.009	**−0.110**	**−0.066**	−0.006	1										
6. MA	**0.108**	**0.199**	**0.079**	−0.035	**−0.086**	1									
7. Tenure	**0.088**	**0.099**	**0.293**	−0.026	**−0.169**	**0.142**	1								
8. Dual	0.013	−0.033	**0.156**	0.003	0.031	**0.060**	**0.113**	1							
9. Size	0.029	**0.258**	**0.249**	−0.021	**−0.107**	**0.343**	**0.130**	**−0.108**	1						
10. Lev	0.006	**0.074**	**0.076**	0.026	**0.080**	**0.190**	**0.042**	**−0.112**	**0.557**	1					
11. CF	**0.053**	**0.165**	**0.060**	−0.027	**−0.120**	0.014	**0.069**	0.007	0.035	**−0.211**	1				
12. Boardsize	0.033	**0.145**	−0.009	**0.067**	**−0.153**	**0.076**	0.013	**−0.204**	**0.187**	**0.118**	**0.072**	1			
13. State	**−0.048**	**0.122**	**0.147**	−0.025	−0.020	**0.044**	**−0.059**	**−0.143**	**0.310**	**0.141**	**0.042**	**0.180**	1		
14. F_Age	**−0.063**	**−0.081**	**0.201**	**0.057**	0.040	**0.065**	**0.098**	**−0.102**	**0.325**	**0.240**	−0.007	**0.117**	**0.068**	1	
15. MShare	−0.004	**−0.114**	−0.015	0.008	−0.010	**−0.053**	**−0.047**	**0.228**	**−0.363**	**−0.329**	−0.027	**−0.213**	**−0.263**	**−0.337**	1

Correlations significant at a level below 10 percent (two-tailed) are in bold.

**Table 3:** Regression analysis of the celebrity CEOs—CSR performance relationship and moderators.

	CSR				
	Model 1	Model 2	Model 3	Model 4	Model 5
Winner	3.827∗∗∗	3.731∗∗	3.700∗∗∗	2.182∗∗	3.751∗∗∗
	(4.24)	(4.13)	(4.09)	(2.24)	(4.13)
CEO_Age		−0.227∗∗			
		(-2.28)			
Winner∗CEO_Age		0.026∗			
		(1.68)			
CEO_Male			0.354		
			(0.16)		
Winner∗CEO_Male			2.186∗∗		
			(2.81)		
ED				0.106	
				(0.01)	
Winner∗ED				6.555	
				(0.48)	
MA					0.649
					(1.62)
Winner∗MA					1.020∗∗
					(2.45)
Tenure	0.262∗	0.444∗∗	0.314∗∗	0.239∗	0.284∗∗
	(1.96)	(3.32)	(2.58)	(1.87)	(2.29)
Dual	−2.358∗	−2.211∗	−2.877∗∗	−2.637∗	−2.976∗∗
	(-1.84)	(-1.66)	(-2.23)	(-1.80)	(-2.30)
Size	6.057∗∗∗	5.716∗∗∗	5.479∗∗∗	7.834∗∗∗	5.428∗∗∗
	(7.28)	(6.83)	(6.56)	(7.98)	(6.43)
Lev	−6.604∗	−6.880∗	−7.349∗	−17.673∗∗∗	−7.439∗
	(-1.70)	(-1.77)	(-1.89)	(-3.87)	(-1.90)
CF	28.773 ∗∗∗	28.125∗∗∗	28.360∗∗∗	24.234∗∗∗	27.959∗∗∗
	(4.88)	(4.80)	(4.84)	(3.67)	(4.73)
Boardsize	1.296	2.280	1.426	−2.048	2.268
	(0.36)	(0.64)	(0.40)	(0.94)	(0.63)

	CSR				
	Model 1	Model 2	Model 3	Model 4	Model 5
State	−5.314∗∗	−4.745∗	−4.705∗	−3.965	−4.824∗
	(-2.00)	(-1.80)	(-1.78)	(-1.39)	(-1.82)
F_Age	−45.780∗∗∗	−42.707∗∗∗	−43.010∗∗∗	−62.143∗∗∗	−43.168∗∗∗
	(-15.83)	(-14.58)	(-14.86)	(-17.00)	(-14.75)
MShare	0.384	1.040	1.122	6.645	0.953
	(0.07)	(0.20)	(0.21)	(0.94)	(0.18)
Year dummy	Yes	Yes	Yes	Yes	Yes
Industry dummy	Yes	Yes	Yes	Yes	Yes
Constant	22.184	28.277∗	26.179∗	41.135∗∗	23.321∗∗∗
	(1.43)	(1.85)	(1.71)	(2.34)	(1.50)
Observations	2141	2140	2140	1822	2121
Adjusted R^2^	0.154	0.170	0.169	0.220	0.171
F-Value	5.61∗∗∗	5.51∗∗∗	5.54∗∗∗	5.49∗∗∗	5.48∗∗∗

∗∗∗p < 0.01, ∗∗p < 0.05, ∗p < 0.1, *T*-statistics are reported in parentheses.

**Table 4:** Robustness tests of the celebrity CEOs—CSR performance relationship and moderators.

	CSR_MScore			CSR	L2. CSR
	Model 6	Model 7	Model 8	Model 9	Model 10
Winner	0.446∗∗∗	0.431∗∗	0.426∗∗	3.733∗∗∗	3.121∗∗
	(3.53)	(3.41)	(3.37)	(4.16)	(3.30)
CEO_Age		−0.022			
		(-1.61)			
Winner∗CEO_Age		0.006∗∗			
		(2.72)			
CEO_Male			−0.101		
			(-0.33)		
Winner∗CEO_Male			0.330∗∗		
			(3.02)		
MA_Content				1.838∗∗∗	
				(3.55)	
Winner∗MA_Content				1.358∗∗	
				(2.95)	
Tenure	0.028∗	0.036∗	0.025	0.250∗∗	0.330∗
	(1.67)	(1.93)	(1.49)	(2.05)	(2.59)
Dual	−0.275	−0.210	−0.282	−2.753∗∗	−2.263
	(-1.52)	(-1.13)	(-1.56)	(-2.15)	(-1.61)
Size	−0.57 ∗∗∗	−0.589 ∗∗∗	−0.599∗∗∗	4.933∗∗∗	6.954∗∗∗
	(-4.94)	(-5.02)	(-5.12)	(5.87)	(7.36)
Lev	0.580	0.609	0.562	−7.472∗	−10.371∗∗
	(1.06)	(1.12)	(1.03)	(-1.93)	(-2.41)
CF	1.706∗∗	1.603∗	1.632∗∗	27.997∗∗∗	34.177∗∗∗
	(2.08)	(1.96)	(1.99)	(4.81)	(5.30)
Boardsize	0.867∗	0.917∗	0.845∗	1.819	1.570
	(1.74)	(1.83)	(1.70)	(0.51)	(0.41)
State	0.110	0.136	0.131	−4.365∗	−4.923∗
	(0.30)	(0.37)	(0.35)	(-1.66)	(-1.66)
F_Age	−10.58∗∗∗	−10.329∗∗∗	−10.433∗∗∗	−42.322∗∗∗	−58.045∗∗∗
	(-26.28)	(-25.19)	(-25.69)	(-14.67)	(-17.07)

	CSR_MScore			CSR	L2. CSR
	Model 6	Model 7	Model 8	Model 9	Model 10
MShare	−1.549∗	−1.482∗∗	−1.509∗∗	0.140	3.258
	(-2.08)	(-2.00)	(-2.03)	(0.03)	(0.53)
Year dummy	Yes	Yes	Yes	Yes	Yes
Industry dummy	Yes	Yes	Yes	Yes	Yes
Constant	62.689∗∗∗	63.155∗∗∗	62.931∗∗∗	25.683∗	36.684∗∗
	(29.26)	(29.44)	(29.32)	(1.69)	(2.14)
Observations	2141	2140	2141	2138	1914
Adjusted R^2^	0.559	0.561	0.561	0.178	0.208
F-Value	6.24∗∗∗	6.12∗∗∗	6.16∗∗∗	5.45∗∗∗	5.31∗∗∗

∗∗∗p < 0.01, ∗∗p < 0.05, ∗p < 0.1, *T*-statistics are reported in parentheses.

Models 2–5 in Table 3 report the results of the moderating effects on CSR performance. We included the interactions between celebrity CEOs (*Winner*) and moderating variables progressively. All interaction terms were mean-centred to avoid potential multicollinearity issues [83]. In Model 2, the interaction between celebrity CEOs (*Winner*) and CEO age (*CEO_Age*) is positive and statistically significant (*β* = 0.026*, p* < 0.1). This indicates that celebrity CEOs’ CSR performance increases with age, thereby supporting Hypothesis 2. In Model 3, the interaction term between celebrity CEOs (*Winner*) and CEO gender (*CEO_Male*) is positive and significant (*β* = 2.186*, p* < 0.05). This suggests that the effect of celebrity CEOs on CSR performance strengthens when the CEO is a male, which is consistent with Hypothesis 3. In Model 4, the interaction term between celebrity CEOs (*Winner*) and environmental dynamism (*ED*) is positive but not statistically significant. Although the sign of the coefficient is consistent with our prediction, we did not find statistical support for Hypothesis 4 in our sample. In Model 5, the interaction term between celebrity CEOs (*Winner*) and media attention (*MA*) is positive and significant (*β* = 1.020*, p* < 0.05). This suggests that the effect of celebrity CEOs on CSR performance strengthens when more media reports on firms have been published, which is consistent with Hypothesis 5.

In summary, our results are generally consistent with our hypotheses. The main effect of the relationship between celebrity CEOs and CSR performance (Hypothesis 1) is supported. Additionally, the moderating effects of CEOs’ characteristics, age, and gender were observed, predicting better CSR engagement (Hypotheses 2 and 3). We also found support for the moderating effect of media attention, predicting positive CSR performance (Hypothesis 5). However, the moderating effect of environmental dynamism did not predict CSR performance (Hypothesis 4).

### 4.2 Robustness tests

We conducted robustness tests to verify the reliability of our results. The results are presented in Table 4. Alternative variables are commonly used to test model robustness in regression analyses. Therefore, the results of using the ‘*CSR Overall Market Rating Score*’ (*CSR_MScore*) in the *Hexun* database as the dependent variable are presented in Models 6–8. The results of using the total number of online financial news with the name of company *i* appearing in the news content in year *t* to measure media attention (*MA_Content*) as the moderating variable is presented in Model 9. The coefficients of celebrity CEOs (*Winner*) and the interaction term between celebrity CEOs (*Winner*) and the moderating variables are positive and significant, suggesting that our results are robust to alternative measures of both CSR and media attention.

Moreover, lagged explanatory variables are commonly used to test robustness in regression analyses. Considering potential endogeneity issues among variables and that the influence of celebrity CEOs on CSR behaviour is unlikely to undergo significant short-term changes, we incorporated a lag of one period for the CSR performance (*CSR*) variable in the original regression model. To further examine the model's effectiveness, we conducted a second robustness test of the original model by lagging the CSR performance (*CSR*) variable by two periods. As shown in Model 10, the estimated coefficient on celebrity CEOs (*Winner*) is positive and significant (*β* = 3.121, *p* < 0.05), indicating that celebrity CEOs are positively related to CSR performance, confirming support for Hypothesis 1.

## 5 Discussion

Using the upper echelons, impression management, and identity theories, this study uncovered the mechanisms between celebrity CEOs and CSR performance and revealed the boundary conditions and underlying mechanisms of celebrity CEOs in influencing CSR. As research on celebrity CEOs is at an exploratory stage and has received less attention from scholars than other types of managers, this study expands the research on celebrity CEOs within the context of China, enriching the examination of the relationship between CEO characteristics and CSR strategies.

First, celebrity CEOs can improve their CSR engagement, which affirms that CSR engagement is an effective approach to celebrity CEOs' impression management focused on protecting and maintaining their celebrity identity. The media represents a potential pressure point for celebrity CEOs; CSR activities are an effective impression management strategy within an objective media discourse. Previous research has indicated that individuals' publicity influences their impression motivation (Leary & Kowalski, 2009). The media, akin to a camera, is perceived by celebrity CEOs as an invisible force of ‘others’ presence’, thereby prompting more positive behaviour in aiding a positive image. Driven by a higher level of self-actualisation needs and the advantages gained by glorified CEOs, they have strong aspirations to maintain their celebrity identity. Nevertheless, celebrity identities are temporary; celebrity CEOs face both identity maintenance and recognition pressures. Thus, with strong impression management motivation to protect their celebrity identities, celebrity CEOs tend to construct positive impressions by engaging in CSR activities.

Additionally, our study revealed the following insights on CSR engagement: 1) Management research does not specify celebrity CEOs' roles. However, our results suggest that responsibility for stakeholders' well-being is and should be embedded in society's expectations of celebrity CEOs' role and identity; 2) As an invisible presence of others, the media encourages corporate executives to exhibit more active and positive prosocial behaviours such as CSR engagement; and 3) CSR can serve as a reputation replacement effect to compensate for poor corporate financial performance.

Second, this study analysed the moderating effects of CEO demographic characteristics (gender and age) and external environmental factors (environmental dynamism and media attention) on the relationship between celebrity CEOs and CSR engagement. It uncovered the critical contingent conditions of the relationship between celebrity CEOs and CSR, offering new contextual explanations for understanding the level of impression management motivation among corporate managers. It also provides new theoretical explanations for identifying and understanding the impression management behaviours and motivations of corporate managers.

Prior studies have suggested that the effectiveness of impression management strategies has boundaries, with the same strategy yielding different outcomes depending on the implementer and context of use ([44]; Graffin et al., 2011). However, prior research has treated the effectiveness of impression management strategies as an incidental conclusion [44,84]. Therefore, the exploration of the boundary conditions of impression management in this study not only supplements the shortcomings of the aforementioned research but also represents an innovative exploration of the effectiveness of impression management strategies of managers.

Moreover, previous studies that have focused on individual-level impression management are relatively mature and extensive, whereas discussions on organisational-level impression management remain in the developmental stage. For celebrity CEOs, what is intriguing is that the motivation behind their impression management can be self-serving. Managers tend to shape their managerial image through altruistic corporate behaviour, and celebrity CEOs’ reputation and image are equivalent to those of the company. Therefore, this study addresses both personal and organisational aspects of CEO impression management by integrating these relationships.

Furthermore, previous studies have suggested that companies engage in impression management as a crisis response, activating it only when negative events occur [44]. However, our conclusions offer a new explanation. Impression management can not only serve as a ‘remedy afterward’ but also as a ‘prevention in advance’, serving as a motivational tool for managers to maintain positive goals. To some extent, this helps enrich the positive understanding of impression management.

We proposed four hypotheses based on the main effect model to examine whether boundary conditions strengthen the relationship between celebrity CEOs and CSR performance. We conclude that CEOs' characteristics—both age and gender—reinforce celebrity CEOs' CSR participation. Our results show that older celebrity CEOs are more likely to improve CSR engagement, suggesting that ageing may influence corporate executives’ physical, cognitive, and learning abilities, can further influence their decision-making processes and lead to organisational risks such as a downturn in financial performance. Under this situation, celebrity CEOs tend to experience a gap between their expected and actual impressions. Consequently, they tend to increase their CSR engagement to compensate for this impression gap.

Moreover, male CEOs can achieve better CSR performance than female CEOs. Owing to the psychological mechanism of status pursuit formed by human evolution, male leaders have a stronger desire for higher status and success. This intrinsic psychological motivation changes the value of celebrity CEOs' impression management goals for identity protection. Therefore, CSR engagement is an effective strategy for male CEOs to maintain their celebrity identity. Here, we would like to offer additional explanations on the gender ratio of managers within firms. Notwithstanding the strides made by Chinese corporations in fostering gender diversity, data from a 2021 report by Morgan Stanley Capital International (MSCI), a leading US-based index provider, reveals that female occupied the CEO position in merely 6.4 % of China's listed companies.^3^ Nevertheless, when juxtaposed with their male counterparts, women's representation in top-tier corporate leadership roles remains disappointingly low. The imbalance concerning the ratio of women in CEO positions, as captured in our research data, objectively reflects the persistent reality of gender disparity in top leadership roles. The push for greater gender diversity in the upper echelons of business continues, reflecting an ongoing commitment to achieving parity.

Regarding the two external environmental factors, only media attention can drive CSR improvement. Overly focused media attention may increase celebrity CEOs' transparency and impose extra supervision pressure on celebrity CEOs. Additionally, owing to the media agenda-setting function, frequent media attention reinforces the public's beliefs on the attribution logic of celebrity CEOs, deepening the public's expectations of celebrity CEOs' roles and increasing their role identity pressure. Thus, celebrity CEOs have an increased desire to improve their CSR engagement to balance identity impressions.

However, environmental dynamism does not affect the relationship between celebrity CEOs and CSR performance as predicted. In other words, environmental fluctuations and corporate executives' uncertain perceptions caused by dynamic environments do not change the degree of celebrity CEOs' impression management motivation. We believe that this is due to the following reasons. On the one hand, reinforcement of a celebrity halo results in CEO overconfidence. This may lead celebrity CEOs to develop an optimistic attribution style in which potential business failures in a dynamic environment are attributed to uncontrollable external factors instead of themselves. Building on McGill's [85] Attributional Style Questionnaire, managers' attribution styles are classified into optimistic and pessimistic. Optimistic attributors tend to attribute business failures to unstable and uncontrollable external factors, remain undeterred by setbacks, and swiftly recover from the shadows of failure. Therefore, poor financial profits may not discourage them and they may not feel threatened by the impression gap. On the other hand, the attribution methods of the main stakeholders related to corporate operations help celebrity CEOs maintain their sense of identity. Additionally, groups have a ‘group-serving bias’ attribution, which also tends to attribute business failures to the external environment [86]. Therefore, in a dynamic environment, celebrity CEOs do not clearly perceive a threat to identity protection; thus, there is no significant change in CSR investments.

## 6 Conclusions

We examined how celebrity CEOs, driven by the goal of protecting and maintaining their celebrity status, influence CSR behaviour by capturing their psychological characteristics. We offer new perspectives on CSR antecedents based on CEOs by providing new exploratory space for motivation research within the managerial psychology dimension.

This study has several practical implications. First, celebrity CEOs care about and value their celebrity identity and aim to protect it through impression management approaches. This shows that individuals' action patterns often reflect their true needs, fulfilling the respect and self-actualisation demands that are an instinctive attribute of human beings as social animals. Meanwhile, our study provides specific and useful methods for celebrity CEOs to protect their identity and reputation. CSR engagement is an efficient and effective impression management strategy, which not only responds to the external expectations of celebrity CEOs’ role in caring for the well-being of stakeholders but also compensates for decline in corporate financial performance.

Second, our ideas also have implications for celebrity CEO's organizations. Organisations generally benefit from the associations with star CEOs, both in the contribution to CSR performance and resource acquisition. The desire for celebrity CEOs to maintain their identity and status can drive more participation in strategic CSR decisions, which can broaden their access to firm resources by utilising celebrity CEOs as scarce and valuable intangible assets for organisations. Organisations can cultivate and utilize celebrity CEOs as valuable intangible resources, and actively strive for celebrity status for CEOs through various media platforms.

Further, we would be remiss if we failed to note the implications of our ideas for authoritative media and media prizes themselves. Our work emphasizes not only the positive role that the authoritative media in creating celebrity CEOs as role models in organisations, but also the key sites of having a clear understanding of celebrity CEOs’ identities or being able to answer the question “who we are”. Therefore, reputable media (including social media outlets) and related media regulators should work together to establish and improve the media award system, so that the media can play a positive role in the business field.

Our study also has some limitations. First, we used secondary data to verify our hypotheses. Although archival databases are objective and reliable, secondary data are not generated for specific research questions, making these data sources inaccurate or unupdated. For instance, in our study, the measurement of CSR performance was obtained from the *Hexun* database. However, there is a lag or incomplete update of recent annual data in this database, making it impossible to conduct a complete analysis of the data. To some extent, this affects the timeliness of our research results. We believe that in future research, secondary data can be used in combination with case studies to explore theoretical ideas through qualitative research, and secondary data can serve as a test method for new theories (eg. Ref. [87,88]), so as to further enhance the credibility and integrity of celebrity CEO research.

Second, variables' measurements and test method can be improved. For example, the measurement of celebrity CEOs in this study is primarily based on whether CEOs received authoritative media awards. Although this measure has been commonly used by scholars, it has some shortcomings. For instance, it overlooks the influence of the public on celebrity CEOs. Kim and Lee [89] applied text analysis to enrich the measurement of celebrity CEOs by measuring the positive emotions of the audience and media attention towards CEOs, which is an optimisation method future research can draw from. Notably, we only examined the influence between celebrity CEOs and CSR engagement with a time lag of one year (*t+1*); we did not judge the persistent influence of celebrity CEOs' identity. Time is a non-negligible factor affecting celebrity CEOs' perceptions of their identity; media and public attention and enthusiasm towards CEOs may decline over time. With time, the external expectations of celebrity CEOs' role identification will weaken, causing celebrity CEOs to gradually adapt to the reality of this decline. These factors may affect celebrity CEOs’ impression management motivations. We believe that the value and significance of celebrity CEOs may lie more in their long-term impact on the organisation. Extending the timeline to a long-term perspective can enable the examination of whether the celebrity effect is transitory. This may also help avoid potential future risks that celebrity CEOs may create in management practices. Lastly, future research should further deal with the potential endogeneity issue and try replicate approaches by using difference-in-differences analysis or propensity score matching methods.

## Data availability statement

The data that support the findings of this study are available from the corresponding author upon reasonable request.

## Ethical statement

This study did not involve humans or animals and did not conduct any animal experiments, clinical trials, or human experiments.

## Funding statement

This work was supported by the 10.13039/501100012456National Social Science Fund of China (Grant Number: 21&ZD322).

## Declaration of competing interest

The authors declare that they have no known competing financial interests or personal relationships that could have appeared to influence the work reported in this paper.

## Figures and Tables

**Table A1:** Variable definitions.

Variable	Definition
*CSR*	CSR performance, measured using the ‘*CSR Overall Rating Score*’ in the *Hexun* database.
*CSR_MScore*	CSR performance (as the proxy for CSR performance in the robustness test), measured using the ‘*CSR Overall Market Rating Score*’ in the Hexun database.
*Winner*	Celebrity CEO, scaled by a binary dummy variable. The variable takes the value of 1 if the CEO won the prizes in 2019–2020; otherwise, it takes the value of 0.
*CEO_Age*	CEOs' age, measured using the actual age of CEOs at year *t.*
*CEO_Gender*	CEOs' gender, scaled by a binary dummy variable. The variable takes the value of 1 if the CEO is male; otherwise, it takes the value of 0.
*ED*	Environmental dynamism, scaled by sales revenue.
*MA*	Media attention, scaled by the total number of online financial news with the name of company *i* appearing in the news headlines in year *t.*
*MA_Content*	Media attention (as the proxy for media attention in the robustness test), scaled by the total number of online financial news with the name of company *i* appearing in the news content in year *t.*
*Tenure*	CEOs' tenure, measured by the actual tenure of the CEO in year *t.*
*Dual*	CEOs' duality, measured by a binary dummy variable. The variable takes the value of 1 if the CEO also serves as the chairman; otherwise, it takes the value of 0.
*Size*	Firm size, measured as the natural logarithm of total assets.
*Lev*	Inverse of leverage ratio, computed as total assets divided by total liabilities.
*CF*	Cash flow ratio, computed as operating cash flow divided by total assets.
*Boardsize*	Board size, measured as the natural logarithm of the board's total membership.
*State*	State ownership, measured by a binary dummy variable. The variable takes the value of 1 if the firm is stock-holding; otherwise, it takes the value of 0.
*F_Age*	Firm age, measured by the natural logarithm of the current year minus the firm's foundation year plus 1.
*MShare*	Managerial shareholding proportion, computed as total managers' shareholding divided by total shareholding equity.

## Footnotes

1 http://www.hdshuju.cn/archives/11630.html.

2 Shepard A, Fan Jiaying. Why is Just Business Reporting So Important in This Era? [EB/OL]. (2016-07-24) [2022−9−21]. https://www.sohu.com/a/107389347_308379].

3 What does it signify that the proportion of female CEOs in listed Chinese companies exceeds developed countries? https://m.gmw.cn/baijia/2022-03/09/1302835677.html.
